# Incidence of Breast Cancer in Eritrea: A Retrospective Study from 2011 to 2017

**DOI:** 10.1155/2019/8536548

**Published:** 2019-07-01

**Authors:** Lidia B. Medhin, Lia A. Tekle, Daniel T. Fikadu, Danait B. Sibhatu, Samson F. Gebreyohans, Kibrom H. Gebremichael, Tesfamariam M. Halki, Saleh M. Said, Yosief T. Ghidei, Hartmut Lobeck

**Affiliations:** ^1^Pathology, National Health Laboratory, Asmara, Eritrea; ^2^Microbiology, National Health Laboratory, Asmara, Eritrea; ^3^National Blood Transfusion Service, Asmara, Eritrea; ^4^Surgical, Orotta Referral Hospital, Asmara, Eritrea

## Abstract

In Africa, breast cancer closely compares with cervical cancer as the most common malignancy affecting women and the incidence rates appear to be rising. Eritrea is experiencing a growing breast cancer problem, but little is presently known on tumor patterns, breast cancer epidemiology, and risk factors. The main objective of this study is to provide baseline data on breast cancer incidence in both sexes in Eritrea. This study was carried out retrospectively and quantitatively by collecting, abstracting, analyzing, coding, and interpreting data recorded in National Health Laboratory (NHL) using CanReg5 ver. 5.00.35. Extracting and classification of the tumor data was done using topography, morphology together with the ICD-10. To generate the incidence rate for the seven years the Eritrean population dataset was used from the population pyramid net for 2014. After we entered all the data from Pathology department in NHL, data was analyzed using the predetermined and developed built-in analysis tools of CanReg5 software and Microsoft Excel 2010. A total number of 9,403 pathology cases were recorded from 2011 to 2017. Out of these 1,497 cases were confirmed as cytology and histology of breast cases. From 1,497 confirmed breast cases in both sexes, the incidence of benign cases was higher than incidence of malignant cases with the case number of 1, 149, and 348, respectively. Out of the 1,497 cases, 1,447 (96.66%) were females; this included a total incidence cases of female benign and malignant breast cases 1,111 (76.78%), and 336 (23.22%), respectively. In both female and male age group the highest positive cases were found in the age greater than 85. The incidence age standard rate per 100,000 in females and male was 3.3 and 0.2, respectively. In sum, the age standardized incidence of breast cancer was relatively low. However, it is our opinion that the low prevalence may be due to low awareness and a highly centralized screening and diagnostic services. This limits access. Altogether, it is our opinion that breast cancer presents a burden to Eritrean ministry of health.

## 1. Introduction

Breast cancer is common invasive cancer with high mortality worldwide. It affects about 5.03% of women worldwide [1]. Female sex, old age, lifestyle, oral contraceptive, hormone replacement therapy, mutations in the breast cancer susceptibility genes BRCA1 or BRCA2, alcohol intake, hereditary factors, and exposure to chemicals can be named as risk factors. Prevention includes change in life style, maintaining healthy weight, less alcohol consumption, intake of marine omega-3, and soy-based foods. Prophylactic mastectomy (removal of both breasts) helps in people with BRCA1 and BRCA2 mutations. Early detection of breast cancer has better prognosis [2]. Physical examination of the breast, mammography, and further tests like Fine Needle Aspiration and histopathological examination is done for diagnosis of breast cancer. Breast cancer is usually treated with surgery, chemotherapy or radiation therapy, or both. A multidisciplinary approach is preferable. Metastatic cancer has less favorable prognosis [3].

The incidence of breast cancer varies greatly around the world; it is the lowest in less developed countries and the greatest in the more developed countries [4]. Since developing countries are going through rapid societal and economic changes, the shift towards life styles typical of industrialized countries leads to a rising burden of cancers associated with reproductive, dietary, and hormonal risk factors [5]. Incidence rates remain the highest in more developed regions, but mortality is relatively much higher in less developed countries due to lack of early detection and access to treatment facilities. Information on the incidence of breast cancer is essential for planning health measures. At present, information on the frequency or epidemiology of breast cancer in Sub-Saharan Africa is scant. This information may be essential for planning health measures. In Eritrea, the representativeness of existing estimates is largely uncertain partly due to the paucity of data and inconsistency in data collection, an outcome which may be linked to the absence of a cancer registry. The main objective of this study was to provide baseline data on breast cancer incidence in both sexes in Eritrea.

## 2. Materials and Methods

This was a descriptive retrospective study. Data recorded between 2011 and 2017 from the National Health Laboratory (NHL) Asmara, Eritrea, pathology department was used. Pathology department in NHL is the only pathology laboratory in Eritrea making it the main data source. Data was entered in Canreg 5 Ver. 5.00.35 software. Developed by the International Agency for Research on Cancer (IARC) in Lyon, France, the open source software can be used to store, check, abstract, code, and analyze curated information using a built-in analysis tools. In this study, no inclusion restriction was placed on age groupings, ethnic groups, or gender. Data from diseased or surviving patients was also included.

**Figure 1:**
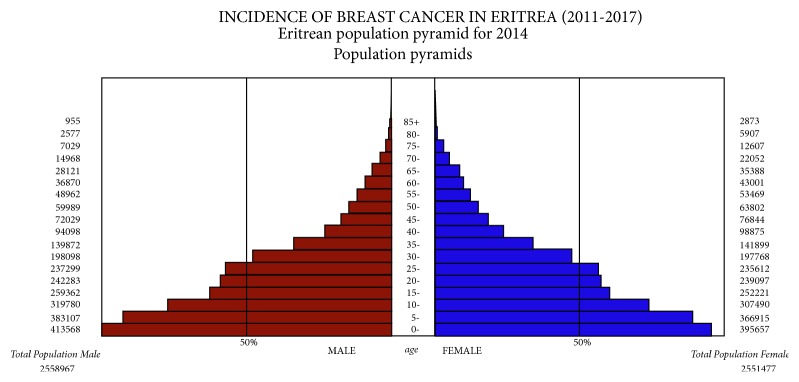
Eritrean population pyramid net 2014 (age-sex distribution).

Eritrean population pyramid net 2014 was used as population data set for generating the ASR per 100,000 (see Figure 1).

### 2.1. Ethical Consideration

Ethical clearance was obtained from the Health Research Ethics and Protocol Review Committee of the Ministry of Health. In addition, all ethical and professional consideration were followed during the study to make patients identity strictly confidential.

## 3. Results

### 3.1. Incidence of Breast Cases in Eritrea

**Figure 2:**
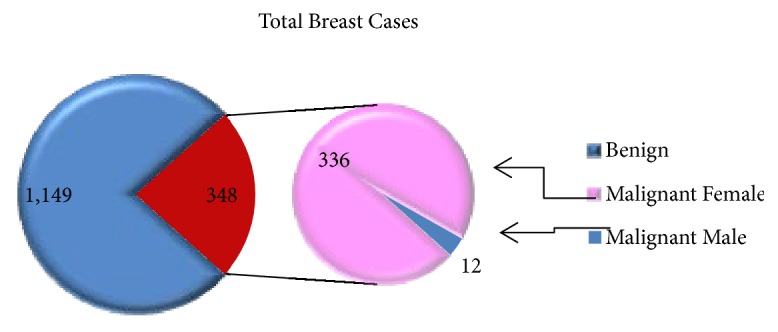
Total breast cases based on behavior from 2011 to 2017.

Generally, 1,497 breast tumor cases were recorded in the pathology department from 2011 to 2017; out of this 348 (23.25%) cases were malignant. From the total malignant cases, 336 (96.55%) were females and 12 (3.45%) were males. From the total of 1,149 benign cases, 1,111 (96.69%) were female and 38 (3.31%) were male cases (Figure 2).

### 3.2. Incidence of Malignant and Benign Cases, from 2011 to 2017

**Figure 3:**
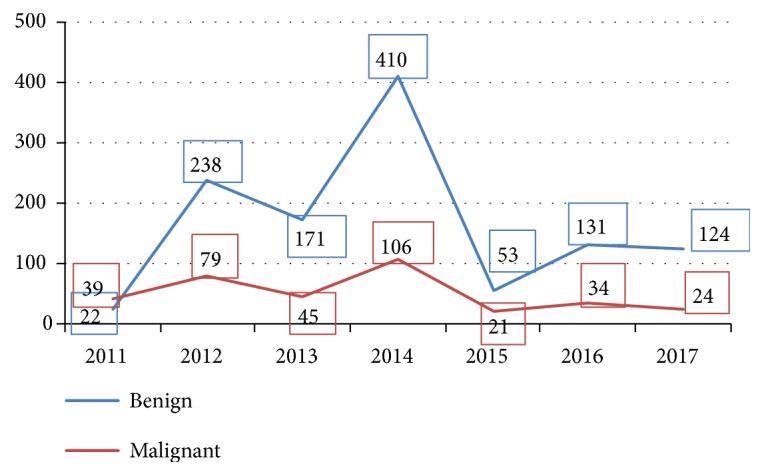
Distribution of Incidence over seven years by malignant and benign tumor cases.

**Figure 4:**
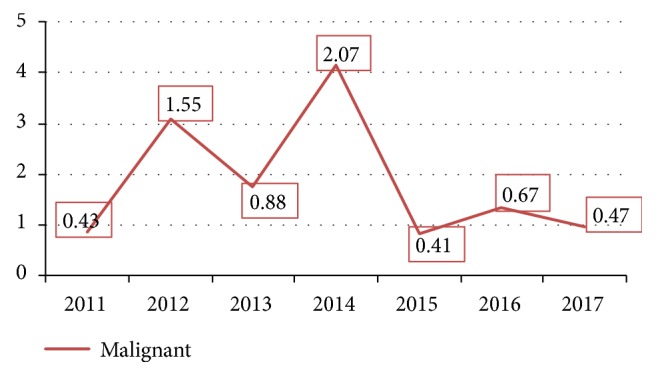
Incidence rates of malignant cases in 100,000 populations over seven years.

Figure 3 represents the number of benign and malignant breast cases and Figure 4 shows the incidence of malignant breast cancer cases over duration of the study. Altogether, the number of benign tumors was consistently higher. In malignant tumors, change in year to year incidence was not significant. However, in benign cases, there was uneven distribution of case numbers, with the cases reaching peaking in 2014 before dropping in 2015.

### 3.3. Incidence Rate (ASR) per 100,000 by Age Group in Both Sexes

The age standardized incidence of breast cancer in Eritrea from 2011 to 2017 is shown in Table 1. The number of males presenting with breast cancer was small. The ASR per 100,000 was 3.3 for females and 0.2 for males. Therefore, the zero values may not capture the true situation on the ground. In females, increasing age was associated with increasing incidence of breast cancer. The highest case seen in females was in the age group above 85 years old and the lowest was seen in the age between 15 and 19 years.

## 4. Discussion

Over the period of seven years study (2011–2017), a total number of 9,403 pathology cases were recorded in pathology department. Out of these, 1,497 cases were confirmed as cytology and histology of breast cases after removed 56 patients duplicated; those patients had the same first name and middle name, but may or may not had similarity in topography/morphology. For these patients exactly duplicated with the same patient and tumor records, we deleted them by leaving the latest incidence date. Therefore, the number of the total recorded patients was reduced from 1,553 to 1,497 cases.

From the total 1,497 breast cases confirmed in the pathology department, benign cases were higher than malignant cases (see Figure 2); this finding is similar to the study done by Fadlelmola et al. where benign cases were higher [6]. In contrast, specific systematic and meta-analytical reviews which leveraged data from population-based registries (36 study sites) estimated that the incidence rates of breast cancer in East Africa were about 28.0 per 100 000 person years in 2018. No regional disparities were observed in the patterns of breast cancer since the pooled rates reported for Sub-Saharan Africa and Northern Africa were 24.0 per 100 000 and 23.2 per 100 000, respectively. Irrespective, the low prevalence of breast cancer reported in this study may be linked to low awareness in the population and limited access to screening services: availability of imaging services (mammography), among others. In addition, the incidence of breast cancer rose sharply between the 4^th^ and 6^th^  (40–60 years) decades of life which accounted for > 60% of all the reported cases. This finding lends credence to reports from Kenya, South Africa, and Ethiopia [7, 8]. But it was lower than reports from Philippines which have 6^th^ and 8^th^ decade, respectively [9]. As it is shown in Figure 3, the number of breast cancer cases (both invasive and noninvasive) from 2012 to 2014 were comparatively higher. The disparity may be due to organized campaigns conducted by the Eritrean government during this period. The drop in 2015 may also be associated with the fact that a full time pathologist was not available at the department in 2015 therefore; a limited number of samples were either referred or diagnosed at the facility. In this regard, the drop in 2015 may actually reflect the inconsistencies in the provision of screening and diagnostic services in the country. This point may partly account for the low incidence rate of breast cancer in Sub-Saharan Africa. Additional factors include lack of reliable cancer registries. Together, these factors point at the possibility that observed variation in the incidence of breast cancer between developed countries and low and medium income countries in Sub-Saharan Africa may not necessarily be due to true differences in risk factors. Moreover, they also point to the possibility that reports in the region underestimate the incidence of breast cancer. The need to increase population level awareness and improve the availability of screening and diagnostic services (screening programs, trainings such as mammography, clinical, and self-examination) is therefore imperative. The lack of resources is also linked to the fact that breast cancer cases are commonly diagnosed at late stages; both palliative and therapeutic treatment are often unaffordable and/or inadequate.

Table 1 demonstrates that incidence of breast cancer is higher in women within > 40 years compared to women under 30 years. Although this result is in keeping with findings from the region [10], the significant number of women under 30 years presenting with breast cancer is notable. The high proportion of women under 30 years of age has also been reported in other studies. This points to the possibility that a proportion of the cancer cases in the region is inherited. Molecular studies targeting the frequency of BRCA 1 and BRCA 2 may help clarify the issue.

## 5. Conclusions

The age standardized incidence of breast cancer was relatively low. However, it is our opinion that the low prevalence may be due to low awareness and a highly centralized screening and diagnostic services. This limits access. Altogether, it is our opinion that breast cancer presents a burden to Eritrean ministry of health. The rising burden of noncommunicable diseases, including breast cancer, may overwhelm the coping capacities of African countries which are currently struggling with infectious diseases. In conclusion, more work should be carried out on the incidence of breast cancer in Eritrea.

## Figures and Tables

**Table 1:** Incidence rate (ASR) per 100,000 of breast cancer in both sexes.

Sex	All Ages	Age Group Structure															CRUDE Rate	%	ASR
		15-	20-	25-	30-	35-	40-	45-	50-	55-	60-	65-	70-	75-	80-	85+			
Female	336	0.3	1.1	1.5	1.7	2.8	5.8	7.3	8.3	7.5	15.9	5.2	6.5	4.5	9.7	19.9	1.8	48.8	3.3
Male	12	0	0	0	0	0.1	0	0.4	0.2	0.9	0.4	0	1.9	2	5.5	0	0.1	27.9	0.2
Cumulative	*348*	*0.3*	*1.1*	*1.5*	*1.7*	*2.9*	*5.8*	*7.7*	*8.5*	*8.4*	*16.3*	*5.2*	*8.4*	*6.5*	*15.2*	*19.9*	*1.9*	*76.7*	*3.5*

## Data Availability

Data will be available upon request from the first author.
